# Default Mode Network Aberrant Connectivity Associated with Neurological Soft Signs in Schizophrenia Patients and Unaffected Relatives

**DOI:** 10.3389/fpsyt.2017.00298

**Published:** 2018-01-08

**Authors:** Liliana Galindo, Daniel Bergé, Graham K. Murray, Anna Mané, Antonio Bulbena, Victor Pérez, Oscar Vilarroya

**Affiliations:** ^1^Neuroimaging Research Group, Neuroscience, Institut Hospital del Mar d’Investigacions Mèdiques, Barcelona, Spain; ^2^Department of Psychiatry, University of Cambridge, Cambridge, United Kingdom; ^3^Cognitive Neuroscience Research Group, Psychiatry and Forensic Medicine, Universitat Autònoma de Barcelona, Barcelona, Spain; ^4^Centro de Investigación Biomédica en Red de Salud Mental, Madrid, Spain; ^5^Cambridgeshire and Peterborough NHS Foundation Trust, Cambridge, United Kingdom; ^6^Institute of Behavioural and Clinical Neuroscience, University of Cambridge, Cambridge, United Kingdom

**Keywords:** schizophrenia, default mode network, connectivity, neurological soft signs, endophenotype

## Abstract

Brain connectivity and neurological soft signs (NSS) are reportedly abnormal in schizophrenia and unaffected relatives, suggesting they might be useful neurobiological markers of the illness. NSS are discrete sensorimotor impairments thought to correspond to deviant brain development. Although NSS support the hypothesis that schizophrenia involves disruption in functional circuits involving several hetero modal association areas, little is known about the relationship between NSS and brain connectivity. We explored functional connectivity abnormalities of the default mode network (DMN) related to NSS in schizophrenia. A cross-sectional study was performed with 27 patients diagnosed with schizophrenia, 23 unaffected relatives who were unrelated to the schizophrenia subjects included in the study, and 35 healthy controls. Subjects underwent magnetic resonance imaging scans including a functional resting-state acquisition and NSS evaluation. Seed-to-voxel and independent component analyses were used to study brain connectivity. NSS scores were significantly different between groups, ranging from a higher to lower scores for patients, unaffected relatives, and healthy controls, respectively (analysis of variance effect of group *F* = 56.51, *p* < 0.001). The connectivity analysis revealed significant hyperconnectivity in the fusiform gyrus, insular and dorsolateral prefrontal cortices, inferior and middle frontal gyri, middle and superior temporal gyri, and posterior cingulate cortex [minimum p-family wise error (FWE) < 0.05 for all clusters] in patients with schizophrenia as compared with in controls. Also, unaffected relatives showed hyperconnectivity in relation to controls in the supramarginal association and dorsal posterior cingulate cortices (p-FWE < 0.05 for all clusters) in patients with schizophrenia as compared with in controls. Also, unaffected relatives showed hyperconnectivity in relation to controls in the supramarginal association and dorsal posterior cingulate cortices (p-FWE = 0.001) and in the anterior prefrontal cortex (42 voxels, p-FWE = 0.047). A negative correlation was found between left caudate connectivity and NSS [p-FWE = 0.044, cluster size (*k*) = 110 voxels]. These findings support the theory of widespread abnormal connectivity in schizophrenia, reinforcing DMN hyperconnectivity and NSS as neurobiological markers of schizophrenia. The results also indicate the caudate nucleus as the gateway to the motor consequences of abnormal DMN connectivity.

## Introduction

Schizophrenia is a severe mental disorder characterized by symptom heterogeneity and frequent impairments in general functioning. A considerable body of research has demonstrated that environmental factors interact with more close-to-genetic factors to modulate the presence of psychotic symptoms, resulting in heterogeneous illness manifestations (1). This heterogeneity makes it difficult to study the neurobiological underpinnings of the illness on the basis of clinical symptoms.

Neurobiological markers and endophenotypes are biological measurable factors that are more closely associated with genetic factors than clinical symptoms; thus, they offer a more solid basis for the study of genetics and therapeutic strategies directed toward specific targets. Common defining criteria for endophenotypes are (a) associated with the illness in the population, (b) heritable, (c) independent of the state of the illness in a particular moment, (d) co-segregated within families with the illness, and (e) also present in unaffected relatives of the patients in higher rates than in the general population (2). The term neurobiological marker is preferred when an association with genetics has not been stated or studied.

Despite the wide variety of studied biological markers in schizophrenia, few studies have performed in-depth analyses of the associations between different types of neurobiological markers or endophenotypes. A positive association between two different candidates should theoretically strengthen their validity as biological markers. Both neurological soft signs (NSS) and brain connectivity have been found abnormal in schizophrenia patients, unaffected relatives and high risk mental states (3–7), pointing them out as neurobiological markers of the illness. However, their correlation has not yet been tested.

One leading hypothesis that has come to the forefront over the past several decades is that schizophrenia is caused by aberrant connectivity between brain regions. The default mode network (DMN) comprises brain regions whose activity is highly correlated and maintained during rest and deactivated when goal-directed behavior is required. Although its activation during resting has been related to mind wandering, self-referential thought, or scene construction, it’s specific role is still controversial, and several studies have reported that the DMN has key roles in a wide variety of cognitive processes (8, 9). Thus, aberrant connectivity within the DMN and between it and other regions may have important consequences for other mental processes such as perspective-taking, self-other judgments (10), and processing of action recognition (11). Interestingly, many of these processes appear to be altered in individuals with psychotic disorders (3). Regions representing the DMN consist of the medial prefrontal cortex (mPFC), posterior cingulate cortex (PCC), precuneus, lateral parietal and temporal cortices, hippocampus, and parahippocampal gyrus (12, 13).

Several studies have demonstrated abnormal connectivity in schizophrenia between different regions of the DMN. While some studies have found reduced resting-state connectivity between the mPFC and temporal regions in schizophrenia, and between the precuneus–PCC and other DMN regions (14–16), many others observed increased connectivity within DMN regions and between the DMN and other regions (3, 4). Overall, the literature contains conflicting results as to the direction of associations. Both significant decreases and increases in functional connectivity (17–19), as well as no significant alterations, have been reported in patients with schizophrenia (20).

Similarly, abnormal connectivity within the DMN has been observed in subjects with first-episode schizophrenia (4), unaffected siblings (3), and individuals at high risk of psychosis supporting the role of abnormal DMN connectivity as a marker of familial vulnerability to schizophrenia (21). These findings show mostly increased connectivity (in the MPFC, bilateral inferior temporal gyrus, precuneus) (5), although an absence of significant differences with respect to controls has also been reported (3, 8, 20).

Neurological soft signs are subtle but observable impairments in simple motor coordination, complex motor sequencing, sensory integration, and disinhibition signs that are neither localized to a specific area of the brain nor characteristic of any specific neurological condition (6, 22) but are associated with deviant brain development (7). It is generally accepted that NSS are more prevalent in schizophrenia patients compared with healthy subjects. They have consistently been demonstrated in neuroleptic-naïve first-episode patients (i.e., before medication exposure), supporting the assumption that NSS constitute an intrinsic feature of schizophrenia (23). Thus, NSS have been suggested as markers of disease vulnerability, which are present before the start of treatment and are independent of the illness state (as well as the type of antipsychotic treatment) (6, 22). Nevertheless, some studies have reported an association of NSS with more chronic and severe forms of the illness (23), with symptom course (24, 25) (lower NSS score with more symptom improvement), and with the duration of untreated psychosis (DUP) (26) (more NSS with longer DUP). Interestingly, NSS have been reported to decrease after initiation of antipsychotic treatment, although more severe baseline NSS predicted a poorer response (27). These results have led some authors to suggest a hypothetical “protective” action of antipsychotics on neurological dysfunction and to qualify the trait vs. state properties.

Recent studies suggesting that schizophrenia spectrum disorders may be characterized by developmental abnormalities in the central nervous system support the notion that NSS may be useful schizophrenia spectrum disorder biomarkers (28). According to this idea, NSS have been frequently reported in schizophrenia spectrum disorders and relatives of patients (29, 30). NSS correlate well between patients and first-degree relatives of the same family (31), and this association has been found stronger in presumed carrier relatives (those parents with a second relative with schizophrenia) vs. non-presumed carriers (32), supporting their status as endophenotypic markers. In particular, motor coordination meets the criteria to be a vulnerability marker of schizophrenia (22). The motor coordination and sensory integration subscales show high heritability estimates based on both the classical heritability equation and genetic model analysis (33).

Although the specific reason for the high prevalence rate of NSS in psychosis is still unknown, a considerable body of recent evidence indicates that NSS are correlated with structural and functional brain abnormalities in schizophrenia (34–37).

This study aims to investigate the association between NSS and the neurofunctional connectivity abnormalities of the DMN in schizophrenia. For this purpose, NSS were evaluated and a functional magnetic resonance imaging (fMRI) was performed in the resting state on a group of patients with schizophrenia, a group of non-affected relatives who were unrelated to the included schizophrenia patients, and healthy controls.

## Materials and Methods

### Subjects

This protocol was developed at the Neuropsychiatry and Addictions Institute of the Parc de Salut Mar de Barcelona between 2012 and 2015. The patients and non-psychotic relatives were recruited from outpatient mental health services and clinics. Control subjects were recruited through advertisements placed in public places. Three groups of subjects were recruited for this study: subjects with a diagnosis of schizophrenia, relatives of subjects with schizophrenia, and healthy controls. Common inclusion criteria for all groups included age between 25 and 50 years, an estimated intelligence quotient (IQ) >80 as measured by Wechsler Adult Intelligence Scale (WAIS) subscales (digit, cubes, vocabulary, and arithmetic, symbol search) (38), living in Spain for more than 5 years and fluency in Spanish. The exclusion criteria for all subjects included the presence of a substance dependence disorder (except nicotine dependence) according to the Diagnostic and Statistical Manual of Mental Disorders, Fourth edition-text revision (DSM-IV-TR), the presence of any other psychiatric disorder besides schizophrenia, or any personal history of severe somatic or neurological disorders.

Specific group inclusion criteria for the group of patients included diagnosis of schizophrenia in their medical records and confirmed by the Structured Clinical Interview for DSM Disorders, disease duration of 5–15 years, treated with atypical antipsychotics, had never received electroconvulsive therapy, and had been clinically stable for the last 6 months [all positive items of the Positive and Negative Scale Symptoms (PANSS), positive subscale scoring 4 or lower] (39).

The non-psychotic relatives were siblings from the same mother and father of a patient diagnosed of schizophrenia according to the DSM-IV-TR, which did not participate in the study (40). Thus, to avoid confounding factors present within families, schizophrenia group participants and non-psychotic relatives participants belonged to different families (for further details, see L. Galindo’s thesis) (41).

The study was approved by the institutional ethics committee. All subjects gave written informed consent and were assured of the confidentiality of the collected data in accordance with the Declaration of Helsinki.

### Clinical Procedures

Basic socio-biographical data were collected from the patients’ medical history, including years of education, socioeconomic level, psychiatric and medical history, years since disease onset, administered treatment, and psychiatric history of first-degree relatives. Patients were clinically assessed using the PANSS (39), and overall subject functioning was assessed using the Global Adaptive Functioning scale (42). IQ was estimated by WAIS subscales (digits, cubes, vocabulary, arithmetic, and symbol search). Pharmacological treatments were compared by calculating equivalent doses of chlorpromazine. To explore the differences between NSS in patients with schizophrenia and their non-affected relatives in comparison to healthy controls, the Neurological Evaluation Scale (NES) (43) was used. The scores of the 28 items were divided in the original subscales of integrative sensory dysfunction, motor coordination, and impaired sequencing of complex motor acts. To facilitate understanding of the test instructions for all subjects, part B of the Rhythm Tapping test (were subjects are asked to produce a series of taps as instructed) was omitted. Two-third year residents in psychiatry were trained by experienced faculty members to perform the NES assessment. The inter-rater reliability was established by the two assessors who jointly examined 20 independent subjects. The intra-class correlation coefficient (SPSS: two-way Mixed Effect Model, confidence interval = 95%) was 0.90 (0.77–0.95).

### Neuroimaging Procedures

To explore the abnormalities in the connectivity of the DMN, a 15-min brain fMRI scan was conducted in resting state. Images were acquired in a Philips Achieva 3T scanner. T1-weighted images were obtained using a fast-spoiled gradient echo sequence [repetition time (TR): 8.2 ms, echo time (TE): 3.7 ms, fractional anisotropy (FA): 88, matrix size: 256 × 256 × 180, voxel size: 0.94 mm × 0.94 mm × 1.00 mm, gap: 0 mm; field of view (FOV): 240 mm]. An echo planar imaging-T2* sequence allowed obtaining the functional volumes, each comprising thirty 3-mm thick slices (TR 3,000 ms, TE: 35 ms, FA: 908, in-plane voxel size 1.80 mm × 1.80 mm, slice thickness 3.0 mm, gap = 1.0 mm, matrix size: 128 × 128, 30 slices; FOV: 230 mm). The acquisition process was completed for 94 subjects. Scanning was voluntarily ended early because of anxiety in one patient with schizophrenia.

### Statistical Analysis

#### Clinical Data Analysis

Clinical and behavioral data were analyzed with SPSS 20 (Statistical Package for Social Sciences for Windows Rel 20, IBM Corp., Armonk, NY, USA). For quantitative data, analysis of variance (ANOVA) was followed by the Bonferroni-corrected *post hoc* tests to perform a between-group mean comparison, whereas chi-squared (χ^2^) tests were performed for qualitative data.

Equal variances in quantitative data between groups were first explored using the Levene test before conducting ANOVA for between-group comparisons. Then, *post hoc* tests were computed using Bonferroni correction, and years of education was included as a covariate since there were significant differences between groups for this variable. We previously computed a minimum sample size of 23 subjects per group to identify a statistically significant difference of greater than or equal to 1 U, considering an alpha risk of 0.05, a beta risk of 0.2 in a two-sided test, an SD of 1.2, and an anticipated dropout rate of 0%.

#### Neuroimage Analysis

Functional magnetic resonance imaging data were preprocessed with the software package SPM8 (Wellcome Department of Imaging Neuroscience, London, UK). Preprocessing was performed with a bespoke script based on SPM and MATLAB functions. After the scripts, 10 random images were also preprocessed manually to confirm the precision of the script.

Image preprocessing and statistical analysis were performed using SPM8 and custom software (spm8w, Dartmouth College) implemented in MATLAB9 (The Mathworks Inc., Natick, MA, USA). First, the anatomical scan was rigid body transformed to match the first functional volume. Functional images were realigned to correct for motion-related artifacts. Subjects with head motion greater than 4 mm translation or 4° rotation in any of the *x, y*, or *z* directions were disregarded.

To correct for between-scan movements, all volumes were realigned to the first volume. Following realignment, coregistration with the anatomical image was performed. Then, functional images were spatially normalized (linear and non-linear transformations) into the Montreal Neurological Institute (MNI) reference system, generating normalization parameters, which were applied to all functional images. For high-accuracy filtering of the images, smoothing with an 8-mm full-width-at-half-maximum Gaussian kernel filter was also applied. For temporal filtering, all data were high-pass filtered (128 s) to remove low-frequency noise.

Three different approaches were followed in the second-level analysis: a region-of-interest (ROI) seed-to-voxel connectivity analysis, an independent component analysis (ICA), and a correlation analysis with NSS score.

Functional magnetic resonance imaging data were analyzed with the software package SPM12 (Wellcome Department of Imaging Neuroscience, London, UK). A general linear model matrix was designed including group, age, and years of study as covariables. The total NSS scores were also included as regressors for further analysis.

#### ROI Seed-to-Voxel Connectivity

To detect neuronal connectivity abnormalities in schizophrenia, the CONN-FMRI Toolbox v1.2 was used to create individual subject seed-to-voxel connectivity maps, to the corresponding seeds of the DMN. First, several brain regions were preselected to represent DMN seeds according to literature review (44, 45). According to the literature, the anterior and posterior regions of the DMN could play different roles in cognitive processes. Within the posterior DMN, the precuneus is thought to be a core functional structure (46), and the mPFC is the most commonly cited region in studies of the anterior DMN. Thus, these two regions were selected as seed regions for further second-level analysis.

Further, the templates available in CONN toolbox, which are a combination of the FSL Harvard–Oxford atlas and AAL atlas cerebellar areas, were used to extract averaged blood oxygenation level-dependent (BOLD) time series for each of these regions. Next, using CONN toolbox, a whole-brain correlation map was obtained for each of the predefined DMN regions and for each subject by computing the correlation coefficients of each seed averaged BOLD time series with the BOLD time course of the whole-brain voxels. These maps were entered in an ANOVA model to evaluate between-group differences. Family wise error (FWE) correction (*p* < 0.01) was used for the main effects of group, and voxel-wise false discovery rate correction at voxel level (*p* < 0.05) was applied for *post hoc* one-to-one between-group comparisons.

#### Independent Component Analysis

Preprocessed images were subjected to spatial ICA, employing the Group ICA fMRI Toolbox (GIFT v3.0a; http://mialab.mrn.org/software/), and was performed in three stages. (1) Principal component analysis (PCA) was employed first to reduce the dataset at subject level to 30 components, and then to reduce it to 20 components at the group level. Subject-level PCA allows preservation of differences between subjects and simultaneously emphasizes similarities between subjects by projecting data into a common space; this allows for the acquisition of mean data for each subject and renders the data computationally tractable. With group-level PCA, data are further reduced into a set number of components and independent group spatial maps. (2) The infomax algorithm was used to decompose the reduced dataset into maximally independent component images. (3) Back reconstruction of the components was performed.

After these analyses, a spatial mask of the DMN available in the GIFT software was applied, and then the components with the maximum spatial correlation with this template were selected as the DMN components of our sample. These components were exported to SPM 12 for the second-level analyses.

To test the effect of group membership on intrinsic functional connectivity of the selected DMN components, one-way ANOVA models controlling for age were conducted in SPM12. A gray matter mask from the SPM was used from as an inclusive mask for all comparisons. Significant main effects of groups were followed up with pairwise comparisons.

Results were interpreted at a voxel-wise threshold of *p* < 0.001 uncorrected and a cluster-wise threshold of *p* < 0.05 FWE corrected, with a minimum cluster size of 50 contiguous voxels. The NES score was correlated with the two components activity.

## Results

### Sociodemographic Characteristics

The study was conducted on 31 patients with schizophrenia, 24 unaffected relatives of patients, and 40 controls. Among them, two patients and two controls were excluded because they did not complete the clinical and neuroimaging procedures. Two additional control subjects were excluded due to either error during fMRI acquisition or the presence of artifacts. In addition, three patients, one of whom had neither completed clinical procedures, one control and one relative were excluded due to exceeding movement threshold during acquisition. The final sample consisted of 85 subjects: 27 patients with schizophrenia, 23 unaffected relatives, and 35 healthy controls. No significant differences between groups were observed regarding age or gender, although patients with schizophrenia had significantly fewer years of education than controls (see Table 1).

**Table 1:** Demographic characteristics in healthy controls, unaffected relatives, and patients with schizophrenia.

	Healthy controls	Unaffected relatives	Patients	*p*

	*N* = 35	*N* = 23	*N* = 27	
Mean age (years) ± SD	36.60 ± 8.0	41.39 ± 10.3	37.60 ± 7.0	0.097
Gender (M/F)	18/17	10/13	16/11	0.538
Mean school level (years) ± SD	12.71 ± 1.8	11.35 ± 2.6	10.0 ± 2.9	<0.05*

*Post hoc analysis revealed significant differences only between patients and controls.

### Neurological Soft Signs

**Figure 1:**
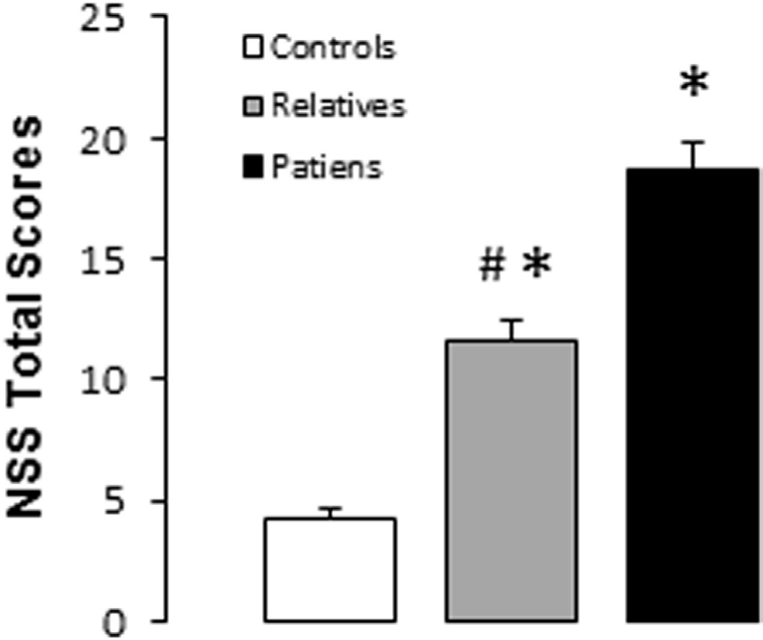
Total neurological soft signs scores in controls, unaffected relatives, and patients with schizophrenia. Bars and error bars represent mean and 1 SD. *p < 0.05 vs. controls; ^#^*p* < 0.05 vs. relatives.

Significant differences were observed between groups for the total and subscale NSS scores (see Table 2). A subsequent *post hoc* analysis revealed significantly higher total NSS scores in both unaffected relatives and patients compared with control subjects. In addition, patients showed higher total NSS scores than unaffected relatives (see Figure 1; Table 2). The subscale analysis (results not shown) revealed significantly higher scores in motor coordination and sequencing of complex motor acts in patients and relatives compared with controls. Patients showed higher scores than relatives in motor coordination but not in sequencing of complex motor acts. For sensory integration, higher scores were observed only in patients relative to the control group.

**Table 2:** Neurological soft signs (NSS) evaluated using the Neurological Evaluation Scale (NES) in patients with schizophrenia, unaffected relatives, and healthy controls.

NSS	Healthy controls	Unaffected relatives	Schizophrenia patients	*F*	*p*

	*N* = 38	*N* = 24	*N* = 29		
Motor coordination, mean (SD)	1.16 (1.05)	2.54 (2.55)	3.83 (2.2)	15.87	<0.001
Sensory integration, mean (SD)	1.18 (0.93)	1.67 (0.92)	3.03 (2.28)	13.27	<0.001
Sequencing of complex motor acts, mean (SD)	0.84 (1.22)	4.29 (1.55)	4.31 (1.81)	60.87	<0.001
Others, mean (SD)	1.5 (1.66)	2.42 (2.0)	5.79 (6.44)	38.79	<0.001
Total NES, mean (SD)	4.68 (3.35)	10.92 (3.87)	16.97 (6.44)	56.51	<0.001

### Neuroimaging Results: ROI Analysis

Table 3 summarizes the regions of the DMN used as seeds, the presence of at least one correlated cluster with significant effect of group, and the between-group *post hoc* comparisons.

**Table 3:** Regions of the default mode network used as seeds with the presence of at least one correlated cluster with significant effect of group and the between-groups *post hoc* comparisons.

Seed	Main effect of group	*Post hoc* comparisons		
		Patients vs. controls	Relatives vs. controls	Patients vs. relatives
Medial prefrontal cortex^a^	Yes	Yes	Yes	No
Posterior cingulate cortex	Yes	Yes	Yes	No
Precuneus L^a^	Yes	Yes	Yes	No
Anterior cingulate cortex R	Yes	No	Yes	No
Anterior cingulate cortex L	Yes	No	No	Yes
Angular gyrus L	No	–	–	–
Inferior parietal lobe R	Yes	Yes	Yes	No
Dorsolateral prefrontal cortex	Yes	Yes	Yes	No
Angular gyrus R	No	–	–	–

*Yes: significant using family wise error correction at cluster level p < 0.01 in the main effect of group or false discovery rate corrected at voxel level p < 0.05 in the post hoc comparisons; no: no significant statistics*.

*^a^Regions selected to show detailed post hoc between-group comparisons*.

Table 4 shows detailed *post hoc* test results for between-group comparisons in the seed-to-voxel connectivity analysis using the mPFC and precuneus as the most representative seed regions of the DMN.

**Table 4:** *Post hoc* between-group comparisons in seed-to-voxel connectivity analysis using regions of interest as seeds.

Seeds	Contrast	Cluster		Peak at MNI (mm)	*k*	Cluster p-FWE
				*x*, *y*, *z*		
Precuneus	P > C	Fusiform gyrus and lateral occipital cortex	L	−56, −62, −6	688	<0.0001
		Left insular cortex	L	−26, 20, 02	503	0.0006
		DLPFC	R	42, 36, −12	353	0.0050
		Superior parietal lobule	L	−26, −52, 42	220	0.0343
		Occipital fusiform gyrus	L	−14, −82, −24	210	0.0482

	R > C	Supramarginal association cortex and dorsal posterior cingulate cortex	L	−14, −44, 44	441	0.0010

Medial prefrontal cortex	P > C	Occipital fusiform gyrus	L	−10, −86, −16	671	<0.0001
		Inferior frontal gyrus, pars triangularis	L	−50, 28, 20	807	<0.0001
		Middle frontal gyrus	L	−36, 00, 52	749	<0.0001
		Middle temporal gyrus	L	−56, −50, −02	670	<0.0001
		Frontal pole	R	52, 48, 10	302	0.0041
		Inferior temporal gyrus	R	58, −56, −12	229	0.0156
		Paracingulate gyrus	R	00, 18, 46	207	0.0238
		Superior parietal lobule	L	−26, −56, 42	174	0.0462

	R < C	Middle frontal gyrus	L	−32, −04, 56	734	<0.0001

*P > C: regions showing increased connectivity in patients with schizophrenia in relation to controls; R > C: regions showing increased connectivity in unaffected relatives in relation to controls; and R < C: regions showing decreased connectivity in unaffected relatives in relation to controls*.

*DLPFC, dorsolateral prefrontal cortex; FWE, family wise error; MNI, Montreal Neurological Institute*.

### Neuroimaging Results: ICA

**Figure 2:**
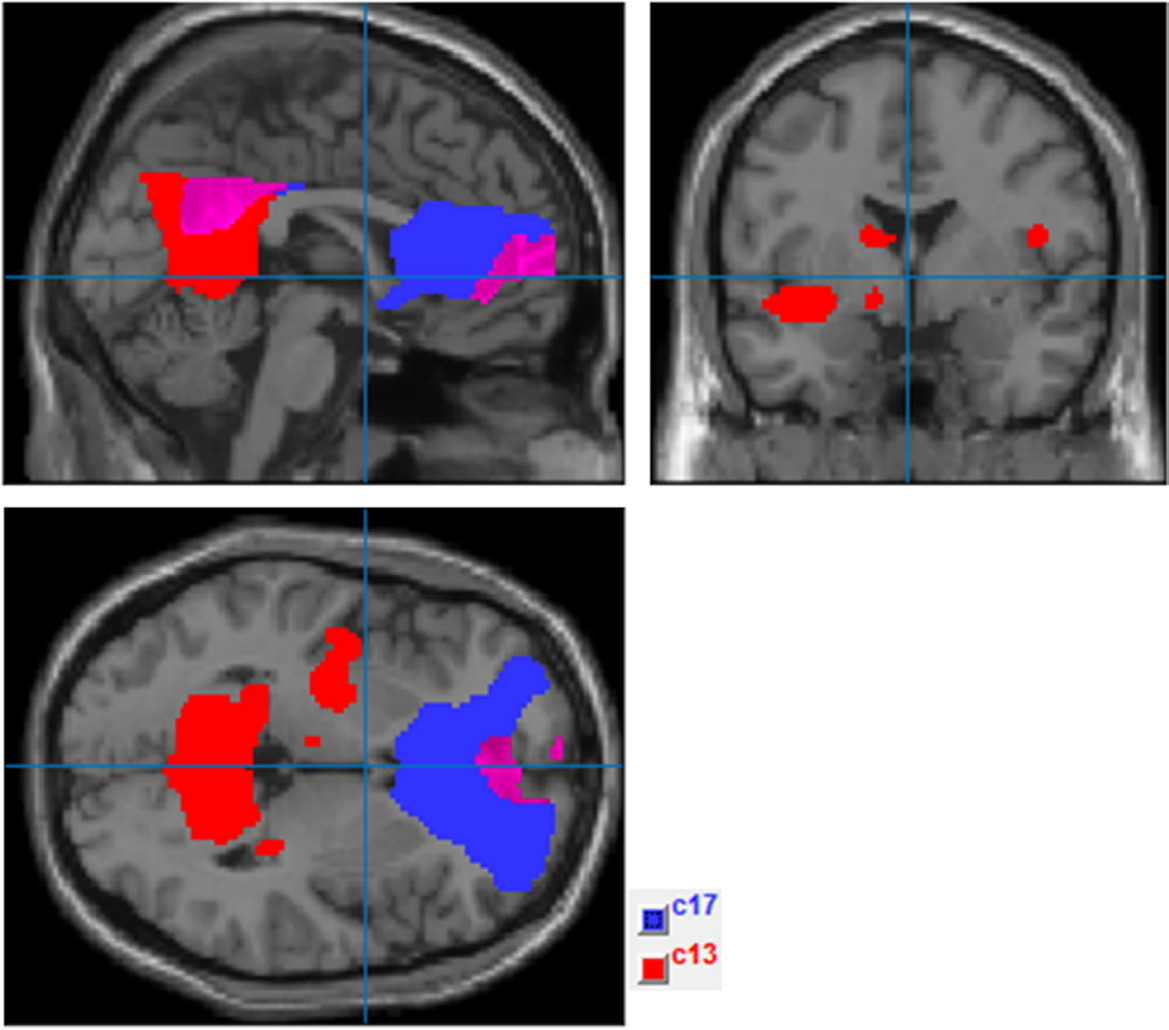
Independent component analysis (ICA) components selected to represent default mode network (DMN) (ComponentsICA.png). This figure shows ICA results after applying the spatial mask of the DMN provided by Group ICA fMRI Toolbox software (http://mialab.mrn.org/software/gift/index.html). Components in blue (c17) and red (c13) were, respectively, selected as the most representative of the anterior and posterior DMN regions.

Two components with a frontoparietal spatial scope were identified as the DMN following ICA. The most anterior component encompassed regions of the mPFC and posterior cingulate/precuneus, and the posterior component included the precuneus, bilateral inferior parietal cortex, left temporal cortex (including the insula and medial temporal regions), and subcortical structures (left thalamus) (see Figure 2).

Table 5 shows the results of the ANOVA model including the mean effect of group and the *post hoc* between-group comparisons. Patients showed significantly increased connectivity in the right PCC in comparison to controls and in the left fusiform gyrus and PCC in comparison to unaffected relatives.

**Table 5:** Independent connectivity analysis: connectivity between-group differences and correlation with NSSs.

Groups	Area	Cluster size	p-FWE	MNI spatial coordinates
				*x*, *y*, *z*
ANOVA (all groups)	Left insula	57 voxels	0.052	40, 0, 4
P > C	R PCC(BA31)	50 voxels	0.036*	22, −59, 29
P > R	L fusiform gyrus	73 voxels	0.019*	−36, −41, 17
	R PCC (BA31)	83 voxels	0.059	12,− 59, 26
	R PCC (BA23)	71 voxels	0.049*	6, −53, 31
R > C	Anterior prefrontal cortex (BA10)	42 voxels	0.047*	26, 46, 17
R < C	Cingulate cortex (BA24)	54 voxels	0.058	−5, 13, 25
	R parietal cortex	103 voxels	0.061	52, −61, 28
	R PCC (BA23)	56 voxels	0.032*	6, −49, 31
Negative correlation with NSS L caudate		110 voxels	0.044*	−14, 6, 18

*Between-group differences in the ANOVA model and post hoc comparisons*.

*P: group of patients with schizophrenia; C: group of healthy controls; R: group of unaffected relatives of patients with schizophrenia*.

*p-FWE, family wise error correction for multiple comparisons; NSS, neurological soft signs; PCC, posterior cingulate cortex; ANOVA, analysis of variance; MNI, Montreal Neurological Institute*.

*p < 0.05.

Also, unaffected relatives showed both significantly increased and decreased connectivity in comparison to controls in different regions of the DMN (see Table 5).

### Correlation between the Connectivity of the DMN and NSS

As shown in Table 5, the NSS scores using NES correlated negatively with connectivity in the left caudate nucleus.

## Discussion

We found different patterns of connectivity within both the anterior and posterior regions of the DMN, ranging from greater to lesser connectivity in patients with schizophrenia, unaffected relatives and healthy controls, respectively. Interestingly, control subjects showed increased connectivity in relation to unaffected relatives in some regions of the DMN. NSS also showed a pattern ranging from higher to lower in patients, unaffected relatives and controls. Importantly, among the DMN components, left caudate nucleus connectivity correlated significantly with NSS.

More specifically, we found increased connectivity in patients with schizophrenia between the precuneus and mPFC seed regions and clusters located at the fusiform gyrus, supramarginal gyrus, somatosensorial association areas, insular regions, and various prefrontal cortex regions. These regions included both intra- and extra-DMN areas.

Thus, we observed predominant hyperconnectivity in the patient group is in line with existing literature. Although early research reported decreased connectivity within and between the DMN (46), several studies have consistently found increased connectivity in patients with schizophrenia, including drug-naive patients and long-term medicated patients (47–49). These results were largely obtained with resting-state fMRI and are also supported by a task performance study that also showed DMN hyperconnectivity (50). Increased connectivity in the DMN of patients while performing a task has been proposed to translate into difficulty deactivating the DMN when activation of other regions is required to perform a task. Some authors have highlighted the robustness of this finding as it is independent of the methodological approach (ROI vs. ICA, resting vs. task) (50). Moreover, hyperconnectivity in the DMN has also been found in unaffected relatives and subjects at high risk of psychosis (47, 49), indicating this feature as a possible neurobiological marker and endophenotype. To date, few studies have focused on the possible association of this DMN hyperconnectivity marker and other candidates to biological markers, which prompted us to investigate the correlation of DMN connectivity and NSS.

As reported previously, we found higher NSS in patients with schizophrenia (6, 29, 51) and unaffected relatives (32, 52) compared with in controls, supporting the hypothesis that NSS is a vulnerability marker for schizophrenia. In addition, these results agree with the idea that NSS segregate with the illness and may be a valid and useful endophenotype (22, 33). The presence of NSS in schizophrenia has consistently correlated with structural atrophy and altered brain activation of predominantly prefrontal and cerebellar cortical regions (36, 53) but also in subcortical regions such as the thalamus, caudate, and globus pallidum (54, 55). These findings have supported the concept of NSS as a manifestation of a generalized brain damage in the cerebello–thalamo–prefrontal brain network. In this regard, we observed a significant correlation between these two candidates to biological markers: NSS and DMN connectivity. Although connectivity of a wide variety of regions correlating with NSS would be expected, the sole finding of the caudate nucleus indicates that this region is a possible gateway between DMN core regions and regions more strongly related to NSS. In other words, abnormal connectivity of the caudate nucleus with other areas that constitute the DMN may underlie the functional and structural abnormalities related to NSS described previously. Interestingly, the role of the caudate nucleus in motor control has been more specifically described as for selecting correct action schemes related to goal-directed action (56). Thus, less connectivity between the caudate nucleus and core brain networks that are predominantly activated in the resting state and are contrary to goal-directed behavior could contribute to the presence of NSS.

The current thinking regarding the etiopathogenesis of schizophrenia points to a “progressive neurodevelopmental disorder” with a developmental predisposition for early degeneration (57). This view postulates disruptions in functional circuits involving heteromodal association areas rather than an abnormality in a single brain region (58). Several studies reported that healthy controls activated prefrontal, thalamic, and cerebellar areas during memory retrieval, but patients with schizophrenia had significantly reduced cerebral blood flow in this circuit (59). The cognitive dysmetria theory states that like motor functions, cognitive abilities are supported by a fluid coordination of activity between the prefrontal cortex, cerebellum, and thalamus [cortico–cerebellar–thalamic–cortico circuit (CCTCC)] (60). This CCTCC feedback loop is thought to monitor and control mental activity involved in many cognitive abilities. In recent years, abnormalities in the CCTCC in task activation (61, 62), resting state (63), and connectivity (64, 65) have been reported in schizophrenia. Our results showing abnormal connectivity in a wide range of regions are in line with this hypothesis. Notwithstanding, caudate nucleus connectivity has been related to reading speed (66), and abnormal caudate connectivity with prefrontal regions has also been found in other psychiatric disorders (67). Among the multiple cortico-subcortical loops involved in brain processes, the caudate nucleus is functionally located at the initial stages, where striatal input signals originate from a broader span of the cortex (68). Considering this, our finding of a correlation between DMN connectivity and NSS through the caudate nucleus is not surprising and reinforces the role of the caudate nucleus as the mandatory passageway between core cortical regions and subcortical and other cortical regions involved in motor control.

The study also faced some methodological limitations. Examiners for the neurological assessment were not blind to group status. Also, there are different neurological scales and subscales to evaluate NSS. We chose the NES because it has been used by the largest number of studies. However, the Cambridge Neurological Inventory or the Heidelberg Scale and Brief Motor Scale are alternative instruments. Further, although the sample size is appropriate for a neuroimaging study, it is not sufficient for many clinical studies. Specifically, the number of participants was too small to analyze patient subgroups.

One strength of the study is that the relatives of patients with schizophrenia included in the study had no familial ties to the patients, thus decreasing the possibility of similar upbringing confounding the results.

All data were high pass (128 s) filtered during image preprocessing to remove low-frequency noise. Although this is a recommended step with seed-based approaches (69), the application of this temporary filter could have affected masking or lowering the power of the results of the GIFT software in the ICA.

In conclusion, our study confirms hyperconnectivity within the DMN of both schizophrenia patients and unaffected relatives, and an association between caudate nucleus connectivity and NSS. These findings strengthen the hypothesis of both DMN connectivity and NSS as neurobiological markers of schizophrenia and indicate a role for the caudate nucleus as the gate to the motor consequences of abnormal DMN connectivity.

## Ethics Statement

This study was carried out in accordance with the recommendations of the Spanish Decree 1090/2015, of 4 December 2015 and Order October 24, 2006 with written informed consent fro all subjects. All subjects gave written informed consent in accordance with the Declaration of Helsinki. The protocol was approved by the Clinical Research Ethics Committee of Parc de Salut Mar—IMIM.

## Author Contributions

LG recruited the subjects and underwent most of the part of the assessments. LG also contributed to the design of the study, developed the most important part of the analysis and wrote a preliminary version of the manuscript. DB contributed to the design of the study, contributed to the recruitment of the subjects, data analysis and conclusions, and has rewritten the manuscript based on LG draft. Also DB is responsible of submitting the manuscript. GM contributed to the discussion of the results and the conclusions. AM contributed to the recruitment and data analysis. AV and VP contributed to study design and development and conclusions. OV contributed substantially to the design and coordinated the development of the study. Also OV contributed to data analysis, conclusions, and the writing of the manuscript.

## Conflict of Interest Statement

DB and AM have received fees as speakers from Otsuka Pharmaceuticals. DB has received fees from Janssen as speaker and specific consultory board for a congress organization. Janssen and Otsuka Pharmaceuticals have covered congress registrations for DB, AM, VP, AV, and LG.
